# Different Roles of Surface Chemistry and Roughness of Laser-Induced Graphene: Implications for Tunable Wettability

**DOI:** 10.1021/acsanm.3c02066

**Published:** 2023-07-10

**Authors:** Alexander Dallinger, Felix Steinwender, Matthias Gritzner, Francesco Greco

**Affiliations:** † Institute of Solid State Physics, NAWI Graz, Graz University of Technology, 8010 Graz, Austria; ‡ The Biorobotics Institute, Scuola Superiore Sant’Anna, Viale R. Piaggio 34, 56025 Pontedera, Italy; § Department of Excellence in Robotics & AI, Scuola Superiore Sant’Anna, Piazza Martiri della Libertà 33, 56127 Pisa, Italy; ∥ Interdisciplinary Center on Sustainability and Climate, Scuola Superiore Sant’Anna, Piazza Martiri della Libertà 33, 56127 Pisa, Italy

**Keywords:** laser-induced graphene, tunable wettability, patterning, high contrast, hydrophobic, hydrophilic, superhydrophobicity, millifluidics, fog basking

## Abstract

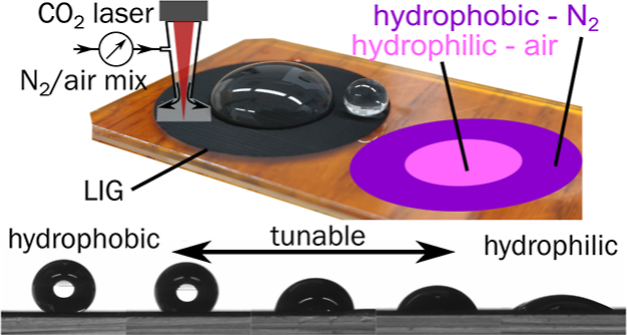

The control of surface wettability is a technological key aspect and usually poses considerable challenges connected to high cost, nanostructure, and durability, especially when aiming at surface patterning with high and extreme wettability contrast. This work shows a simple and scalable approach by using laser-induced graphene (LIG) and a locally inert atmosphere to continuously tune the wettability of a polyimide/LIG surface from hydrophilic to superhydrophobic (Φ ∼ 160°). This is related to the reduced amount of oxygen on the LIG surface, influenced by the local atmosphere. Furthermore, the influence of the roughness pattern of LIG on the wettability is investigated. Both approaches are combined, and the influence of surface chemistry and roughness is discussed. Measurements of the roll-off angle show that LIG scribed in an inert atmosphere with a low roughness has the highest droplet mobility with a roll-off angle of Φ_RO_ = (1.7 ± 0.3)°. The superhydrophobic properties of the samples were maintained for over a year and showed no degradation after multiple uses. Applications of surfaces with extreme wettability contrast in millifluidics and fog basking are demonstrated. Overall, the proposed processing allows for the continuous tuning and patterning of the surface properties of LIG in a very accessible fashion useful for “lab-on-chip” applications.

## Introduction

Laser-induced graphene (LIG) is a 3D porous and conductive carbon material that was first investigated in 2014.^1^ By treating a non-conductive polymer sheet, like polyimide (PI), with radiation of an IR-CO_2_ laser, a photothermal and photochemical process called laser-induced pyrolysis takes place. This process transforms the polymer precursor into LIG.

The first pioneering work of Tour and collaborators laid the basis for further fundamental investigation of the process, structure, and composition of LIG materials. This resulted in an extension to many other synthetic^2−6^ and natural precursors,^7−11^ as well as to other laser sources (e.g., visible and UV).^8,12−18^ Many different applications have been tested, including sensors,^19−21^ energy applications,^22^ and actuators.^23−25^ One of the main benefits of LIG is the precise, fast, and mask-free patterning of conductive circuits over large areas. This can be done with flexible polymer sheets or even bio-derived materials,^26^ without the need for additional treatment or of complex processing facilities. This brings enormous benefits in terms of cost compared to screen, ink-jet printing, or other deposition methods currently adopted in printed and flexible electronics.

The structural and compositional changes in LIG can have drastic consequences on its functional properties. Along with electrical conductivity, the surface energy (and in turn wettability) can vary widely in connection with changes in the LIG composition and morphology. Some studies have evidenced that the wettability of LIG can be altered by different approaches^27−30^ and showed a promising and cheap way to create patterned superhydrophobic/superhydrophilic surfaces. Furthermore, by grafting halogenated chemical groups, even superlyophobic LIG surfaces can be created.^30^ The control of surface wettability is indeed a technological key aspect and usually poses a considerable effort connected to its high cost, nanostructure, and durability.^31^ Many important technical applications depend on hydrophobic surfaces, such as anti-icing, self-cleaning surfaces, anticorrosion, water–oil separation, biomedical applications, and microfluidics.^32^ Inspired by nature, some scientists adopted strategies found in biological structures to achieve this goal. The most prominent and well-known example is the lotus leaf.^33^ With a contact angle of Φ ≤ 170° and a hierarchically structured surface, the lotus leaf can repel water to achieve self-cleaning. Other examples of natural micro-nanostructured surfaces with special wettability are, among others, bird feathers (ducks, pigeons) and insect legs, such as in the case of water striders.^34^ Biomimetic approaches to special wettability have emerged in the last few decades, successfully replicating this behavior in artificial materials through hierarchical structuring and/or fine tuning of surface chemistry.^35^ However, many of these processes are very time consuming and/or use unsustainable halogenated chemicals such as fluorine to lower the surface energy.^30,36^

Different properties of LIG can be tuned by changing the lasering parameters. The most obvious change is the one in morphology. One can tune the morphology from a flat porous LIG structure, called LIG-P, to a dense fiber forest of LIG with a height of several hundreds μm, called LIG-F.^37−39^ This change is related to the laser fluence, which also tunes other properties of the LIG, such as its conductivity and its wettability. The latter has been ascribed to the presence of oxygen and oxygen-containing surface groups on the LIG surface, created during processing in an air environment.^29^

To date, two basic strategies have been investigated to change the wettability of LIG: changing the surface oxygen concentration of the LIG surface by changing the oxygen content of the processing atmosphere (i.e., operating in a closed chamber with an inert atmosphere^29,40^) or by changing the surface morphology to favor Cassie–Baxter or Wenzel states.^27,28,41−43^ However, it has been shown that the surface oxygen concentration of LIG can also be tuned by changing the laser fluence,^28,30^ so the effect of surface morphology is not entirely clear.

The first technique requires placing the precursor within a closed chamber with a controlled H_2_ or Ar atmosphere to eliminate oxygen during the laser-induced pyrolysis; the chamber is equipped with an infrared transparent ZnSe window for enabling laser scribing.^29^ This approach has obvious limitations in terms of large-area patterning, ease, speed, and cost of processing, making it impractical compared to others and limiting its upscaling to industrial processing. Furthermore, LIG is often used for electrochemical sensors where wetting and the amount of oxygen defects on the surface play a critical role in sensing performance. More sensitive sensors can be developed by understanding the relationship between wetting and defects in LIG.^20,44−46^ In this study, two approaches were investigated. First, a new method of modifying the surface oxygen concentration of LIG by so-called nitrogen (N_2_) purging during the laser scribing process was adopted. A local atmosphere with controlled and tunable composition (i.e., oxygen content *c*_O_2__ in % vol, within the full range 0–21% vol) was created directly where the laser beam hit the surface and the laser-induced pyrolysis into LIG took place. This allowed the tuning of the amount of surface oxygen groups on LIG. By tuning the oxygen content *c*_O_2__ in the local atmosphere, we were able to continuously tune the wettability of LIG from superhydrophilic (contact angle Φ < 10°) to (super)hydrophobic (Φ > 150°) in a controlled way. Patterns with extreme wettability contrast could be created over a large area within minutes and without any use of chemicals or complex facilities. The superhydrophobic samples showed no degradation over a period of more than one year and multiple measurements. Furthermore, the influence of surface morphology on wetting behavior was investigated. For this, an approach we discovered for our laser setup was utilized, similar to Nasser et al.^28^ We were able to change the laser spot density (and therefore fluence), which was determined by the so-called “gray value” of the raster scribing pattern, as described in the Materials and Methods. Certain settings resulted in a wetting behavior that could be tuned from superhydrophilic (Φ < 10°) to hydrophobic (Φ ≈ 150°) by just changing the LIG spot density. Also, in this case, extreme wettability and contrast patterning could be easily achieved. Applications in millifluidics and fog basking for this second approach were demonstrated.

To investigate the relative influence of roughness and chemical composition on the wettability of LIG and discriminate the two contributions, we combined both approaches to scribe density patterns with a low content of surface oxygen. A split in wetting behavior for the inert and ambient scribed LIG was observed at a certain LIG density. Furthermore, the dynamic wetting behavior of the samples was investigated by measuring the roll-off angle. Ambient scribed samples showed a higher roll-off angle than nitrogen scribed samples, with a roll-off angle of Φ_RO_ ≈ 1°.

## Materials and Methods

### LIG Synthesis/Patterning

A Laser Cutter/Engraver (Universal Laser Systems VLS 2.30, Power 30 W) operating with a CO_2_ laser source at 10.6 μm wavelength and equipped with an HPDFO (High-Power Density-Focusing Optics) beam collimator (nominal beam size in focus: 25.4 μm) was used to create conductive patterns of LIG onto PI tape (Kapton HN, thickness = 50 μm, with silicone glue, supplied by M&S Lehner GmbH). PI tape was attached to glass microscope slides (25 × 75 × 1 mm, ISO 8037/1, Epredia) for easier manipulation of samples.

These settings of laser rastering parameters were employed for producing a LIG-P: *P* = 10%, *S* = 10%, raster resolution of 500 PPI, image density (ID) of 5 (arbitrary scale, defining *a* spacing between consecutive rastered lines of ∼50 μm), and a positive defocusing of *Z* = 0.7 mm. LIG-F was produced with the same setting as LIG-P except for the power: *P* = 20%.

### N_2_ Purging and Oxygen Measurements

The laser cutter was equipped with a coaxial gas nozzle to blow pressurized air onto the laser spot. The nozzle was used to flow a mixture of N_2_ and air onto the scribing area to tune the oxygen content *c*_O_2__ of the local atmosphere.

The nozzle diameter was drilled out to 4 mm to make the alignment of the cone with the laser system easier. The bottom of the cone was 2 mm above the focus point. The flow rate for pure nitrogen scribing and local atmospheres with reduced oxygen content *c*_O_2__ was set to 5–6 L/min.

For measurement of the oxygen content *c*_O_2__ in the purged air, a DS0122 ZrO_2_ Screw Fit Probe Zirconium Dioxide O_2_ sensor from SST Sensing Ltd. was used. The sensor was connected to a PC using an Arduino with custom code for communication with the DS0058 OXY-LC Oxygen Sensor Interface board (SST Sensing Ltd.) of the O_2_ sensor. The sensor was placed in a custom mixing chamber with a diffuser to avoid direct gas flow onto the sensor’s surface. The gas mixture entered the chamber, was measured, and used to create the local atmosphere. The sensor was calibrated by following the “operating principle and construction guide” found on the manufacturer’s website. For the calibration, compressed air at a pressure of 0.6 bar was used. The total pressure of the gas mixture was held constant and was controlled indirectly via a flow meter. A schematic of the setup can be found in the Supporting Information, Figure S1.

### Density Pattern

The native software of the laser cutter uses a dithering method for translating shades of gray of an original gray-value pattern image into patterns of black dots. This dithering method was used to convert different gray values (*G* = 0%: white and *G* = 100%: black) into a scribing pattern at varying densities of lasering spots. LIG-P and LIG-F samples were scribed with such gray-value patterns for the investigation of their wettability. For a more detailed explanation, see the corresponding section in the Supporting Information.

### Demonstrators

Two proof-of-concept demonstrators of high wettability contrast surfaces were prepared to show the potential use of the proposed approach in millifluidics and fog basking.

For the millifluidic demonstrator, LIG with the density pattern approach was scribed. The PI tape was attached to a microscope glass slide as described before. The hydrophobic border pattern was scribed with LIG-F settings and *G* = 8%. The hydrophilic channels were scribed with LIG-P settings and *G* = 100%. The water for the millifluidic device was mixed with two different fluorescent dyes (rhodamine B and rhodamine 6G). The fluorescent mixtures were exposed to UV light.

For the fog basking demonstrator, samples with different LIGs (LIG-P G30, LIG-F G16, and LIG-F*) and pristine PI were prepared with PI tape on microscope glass slides as explained before. LIG-F* was designed by applying the findings of Garrod et al., where an optimal distance between hydrophilic (pristine PI) and hydrophobic centers (LIG-F G16) was found to be 1000 μm.^47^ The setup for the fog basking experiment consisted of a Rowenta HU5220 Aqua Perfect Silent Humidifier, with the humidity setting at 90% RH and maximum fog. A sample of patterned LIG@PI or pristine PI was placed 4 cm away (45° tilted) from the hose outlet (10 mm in diameter). The “active” collection area was the 25 × 25 mm PI tape which was used to calculate the collection rate. Experiments were carried out at 22 °C with a room RH < 30%. The condensation water was collected in a Petri dish and weighed with a Sartorius LD2200P-00V1 scale every 15 min. Microscope images were taken with a Leica Wild M3B optical microscope, and the videos were recorded with a smartphone camera. A schematic of the setup can be found in the Supporting Information, Figure S2.

### Characterization

Contact angle measurements were conducted using a sessile drop technique, with a setup by KSV Instruments Ltd., using a CAM 200 Optical Contact Angle Meter. A drop of deionized water was placed on the surface of interest, followed by the calculation of the contact angle with the KSV CAM2008 software. The drop volume was kept around 10 μL. Three measurements for each sample were conducted and averaged. Roll-off angles were measured with a custom tilting setup. The sample holder was slowly tilted manually until the droplet (10 μL) rolled off. The setup was limited to measuring roll-off angles up to a value of 90°. A video was recorded of the droplet, and the roll-off angle was evaluated by measuring the angle of the tilted substrate in a graphical software. At least three droplets on three samples were measured for each sample type.

Sheet resistance was measured with a custom 4-point probe setup consisting of a Keithley 2602B source meter and four linearly arranged measurement tips with a distance of *d*_probes_ = 1.5 mm. Measurements were carried out on square samples with a length of 6 mm. The values reported are averaged over 9 measurements and at least 3 samples. The sheet resistance *R*_s_ was calculated with the following equation
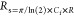
1where *C*_f_ = 0.687 is the correction factor for a finite thin square.

The electron microscope imaging of LIG was performed with a JEOL JSM-6490LV scanning electron microscope, operating at 5–20 kV acceleration voltage.

The Raman spectra were measured using a LabRam HR800 combined with an Olympus BX 41 microscope. The laser wavelength was 352 nm (5 mW); an integration time of 4 s × 4 accumulations, a slit/hole size of 200 μm, a 300 lines/mm grating, and an ×50 LMPlanFLN (NA = 0.5) objective were used. The shown spectra are the average of at least 3 spectra taken at different positions on the sample, they have been background corrected, and the intensity of the G-band has been normalized.

X-ray photoelectron spectroscopy (XPS) measurements were performed in an ultrahigh vacuum (UHV) chamber equipped with a dual anode X-ray source (Al/Mg) and a hemispherical electron energy analyzer (SPECS Phoibos 150). The LIG samples on PI were carefully removed from the glass support and fixed with the help of carbon tape on a Ti sample holder, which was introduced into the UHV chamber via a load lock. The measurements reported in this work were acquired with monochromatic Al Kα radiation (400 W). Both low-resolution survey scans and high-resolution detail scans of the regions of interest (C 1s and O 1s) were taken at normal emission. Quantification of the carbon and oxygen content was performed within the Prodigy software (SPECS).

Attempts at measuring the real surface roughness via optical profilometry were unsuccessful because of the reflective nature of PI with the combination of black/absorptive LIG. The following workaround was used to estimate the change in roughness related to the LIG coverage. The roughness and LIG coverage were estimated via microscope images and a Python script that converted the images into a 2-bit image (black and white) with certain thresholds. The following equation was used to calculate the surface roughness with *f*(*x*, *y*) being the height at position *x*, *y* (0 for PI or 1 for LIG), *f* being the mean height of the sample, and *L* being the sample area
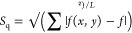
2

## Results and Discussion

### Nitrogen Purging

**Figure 1:**
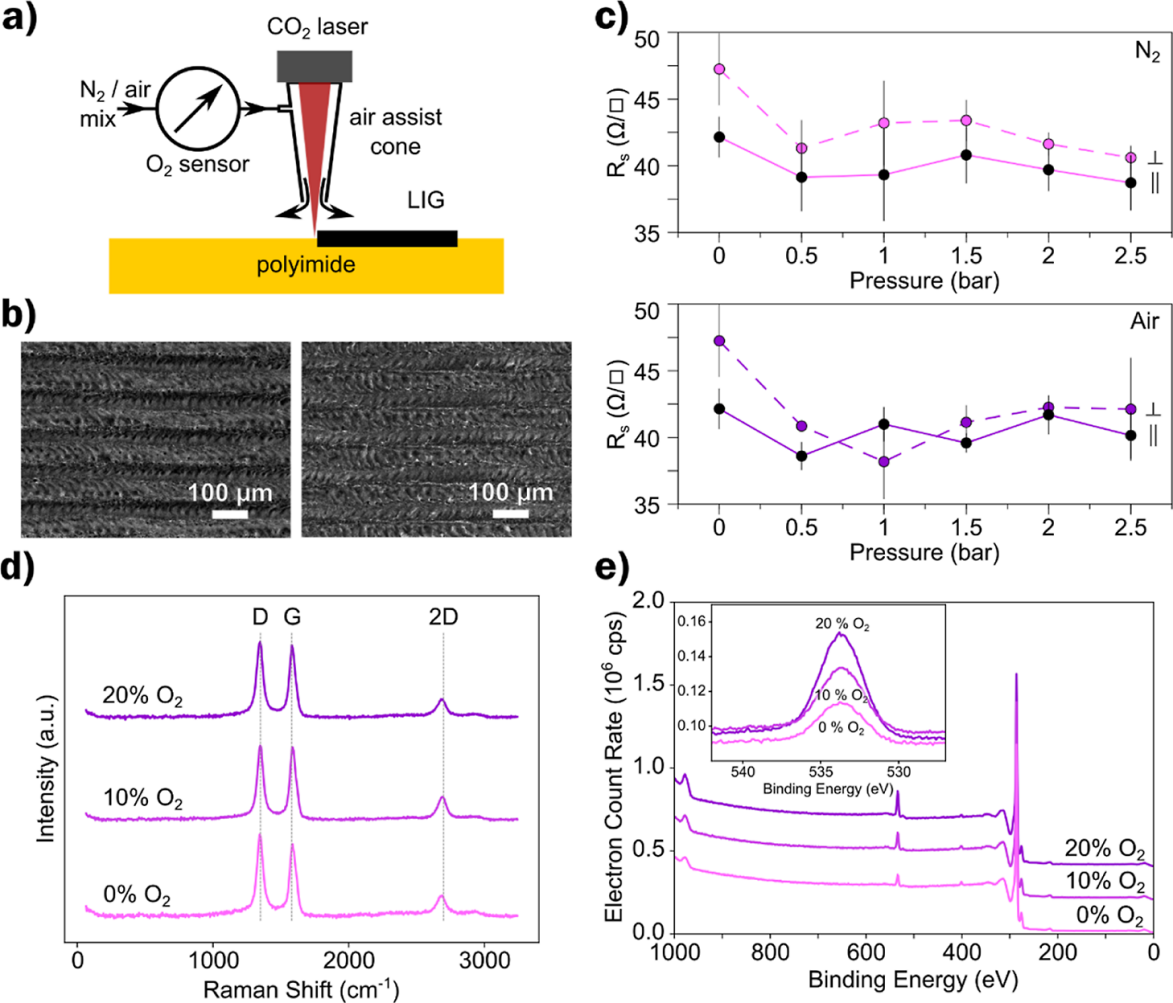
(a) Schematic representation of LIG scribed under a local atmosphere with tunable oxygen content *c*_O_2__ using an air assist cone. (b) SEM images of LIG-P without (left) and with nitrogen purging at 0.5 bar (right). (c) Sheet resistance of LIG-P for different purging pressures with N_2_ (top) and air (bottom) as measured along perpendicular (⊥) and parallel (∥) orientations to the laser scribing direction. (d) Raman spectroscopy of LIG-P scribed under different O_2_ concentrations. (e) XPS for LIG-P scribed under different O_2_ concentrations, inset: detail of oxygen peak.

Instead of using a closed chamber to create an inert atmosphere as shown in other publications,^29,40^ which limits the practicality, a local atmosphere was created in the laser scribing area by using an air assist cone, as shown in Figure 1a. The cone nozzle mounted on the laser lens (moving together with it during rastering) had a circular aperture, concentrically with the laser beam, permitting it to blow the desired gas/air mixture directly on the sample surface. A mixture of nitrogen and compressed air with controlled and tunable oxygen content *c*_O_2__ was purged through the cone and created a local atmosphere just at the laser spot area. This system avoided the need for a closed chamber and discontinuous vacuum/venting operations. Instead, the purging system can be switched on and off in real time, permitting the continuous change of the scribing atmosphere.

First, the morphology and electrical properties of LIG-P scribed in two extreme atmospheric conditions were investigated and compared: pure N_2_ purged (*c*_O_2__ = 0% vol) and air purged (*c*_O_2__ = 20.7% vol, the normal oxygen concentration in air) LIG-P. Images taken by scanning electron microscopy (SEM) show only minimal differences in the morphology of both LIG-P variants (Figure 1b). This is in contrast with the findings of Li et al.^29^ and Mamleyev et al.,^40^ who could observe some morphological differences between air and nitrogen LIG. It can be assumed that this had two reasons: first, the whole process in this case took place in a completely inert atmosphere; second, the gas was introduced into the chamber from the side. The latter also showed to have an effect on the wettability when considering the laser scribing direction.^29^ However, in the case of LIG-F (Figure S3), a difference in the morphology between N_2_ and air is visible. One can see that the fibers got partly destroyed or clustered when scribed with N_2_ purging, which was to be expected given the shape of the fibers. Therefore, for subsequent investigations with nitrogen purging, only LIG-P was considered. A detailed investigation of the morphology and properties of LIG-P and LIG-F scribed just in air is available elsewhere.^37^

The sheet resistance of LIG-P produced in a N_2_ atmosphere was only slightly higher (*R*_s_ = (40 ± 2) Ω/□) than the one of LIG-P produced in the air (*R*_s_ = (45 ± 4) Ω/□). This is in contradiction to the results obtained by Mamleyev et al. where a decrease in sheet resistance was observed.^40^ It is possible that the chamber scribing resulted in LIG-P with a higher nitrogen doping than in the case of just purging. However, Mamleyev et al. did not provide any information on the chemical composition.

No significant influence of N_2_ and air purging pressure on the electrical properties was evidenced in the investigated pressure range of 0–2.5 bar (Figure 1c). Considering that LIG has a scribing direction, which also influences the sheet resistance when measured either perpendicular to or parallel to the scribing direction, both directions showed the same behavior for N_2_ and air.

By mixing N_2_ and compressed air, the oxygen content of the local atmosphere *c*_O_2__ could be changed from 0% vol to around 20.7% vol (ambient condition), and the influence of this parameter in the composition and structure of the resulting LIG-P was investigated by means of Raman spectroscopy and X-ray photoelectron spectroscopy (XPS). Raman spectroscopy of LIG-P species scribed at different *c*_O_2__ levels showed no difference in LIG quality (Figure 1d). The typical bands for LIG were present: D, 2D, and G bands,^1,48^ showing a ratio of *I*_D_/*I*_G_ of 1.08 ± 0.11 and a ratio of *I*_2D_/*I*_G_ of 0.31 ± 0.07 (@ N_2_ purging, *c*_O_2__ = 0%). The values for the intensity ratios for the samples with *c*_O_2__ = 20 and 10% are similar and can be found in Table S1. This indicates LIG with a higher amount of defects and a lower amount of graphene layers compared to that mentioned in other publications.^1,37^ However, since the nitrogen purging did not change the intensity ratios, the LIG can be tuned for fewer defects by changing the other laser parameters.

The elemental composition of the LIG surface for different *c*_O_2__ was measured via XPS. The XPS results are shown in Figure 1e, with an inset showing the detailed O 1s peak. Exact values and XPS spectra can be found in the Supporting Information (Table S2). The spectra show that the concentration of oxygen on the surface of LIG increased from (2.65 ± 0.05) at % for pure N_2_ to (5.18 ± 0.05) at % for LIG scribed in air. This confirms the findings of Li et al.^29^ that the surface properties of LIG are dependent on the atmosphere present during the laser-induced pyrolysis. Furthermore, it shows that the surface properties, mainly the surface oxygen concentration of the surface and the resulting wetting behavior can be tuned by a locally induced atmosphere.

**Figure 2:**
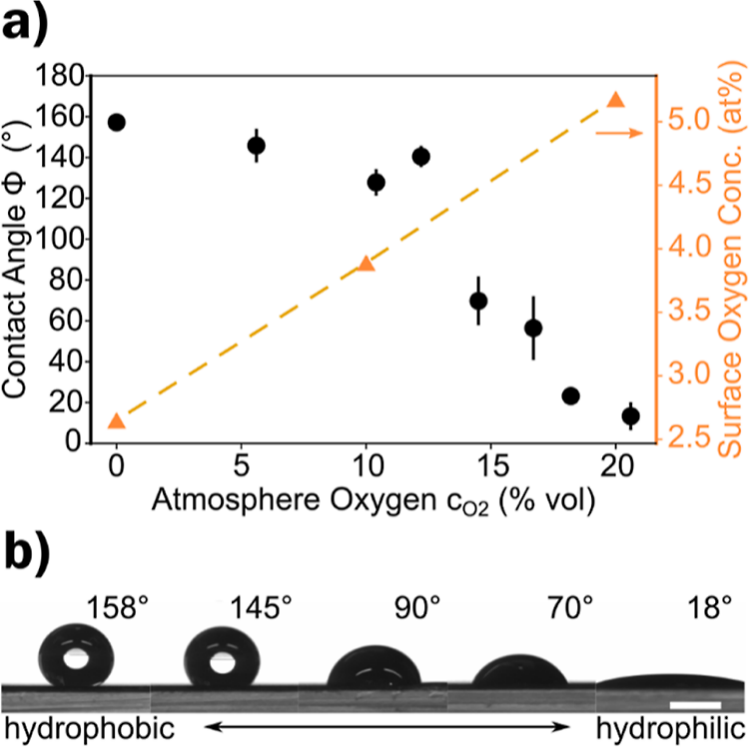
(a) Contact angle and concentration of surface oxygen of LIG plotted versus *c*_O_2__. (b) Images of the contact angle measurements showing the change in wettability for LIG-P at different *c*_O_2__ (from left to right: 0, 12.2, 14.4, 16.7, and 20.7%) and scale bar = 1 mm.

While this change in the elemental composition has only a minor effect on the sheet resistance of LIG-P, its wettability is drastically affected by that. The contact angle of LIG samples scribed at different *c*_O_2__ is plotted in combination with the surface oxygen concentration of LIG in Figure 2a. Samples scribed at *c*_O_2__ < 12% showed a distinct hydrophobic behavior. A superhydrophobic behavior, with a contact angle as high as Φ = (157 ± 1)° was observed for a complete N_2_ atmosphere. This maximum value is the same as observed by Li et al., who measured 157° for LIG created with H_2_ in the chamber.^29^ This further demonstrates that purging is sufficient to create an inert local atmosphere. With increasing oxygen concentration in the local atmosphere, the contact angle decreased to a minimum value of Φ = (13 ± 7)°, evidencing a hydrophilic behavior.

Figure 2b shows the images taken for the contact angle measurements plotted in Figure 2a, one can clearly see the decrease in contact angle with increasing surface oxygen concentration. However, there is no good explanation for the increase in contact angle for 12.5% of oxygen in the local atmosphere. The measurements were reproduced several times and always showed an increased value, not in line with the overall trend. This could maybe be an indicator for a systematic error of the oxygen sensor that is related to the partial pressure of nitrogen or air.

The samples under complete nitrogen scribing (0% oxygen in the local atmosphere) were tested again after one year, and the measurements showed that the hydrophobic nature of the LIG (Φ_1y_ = (156 ± 7)°) was maintained over the whole time.

### LIG Density Pattern

**Figure 3:**
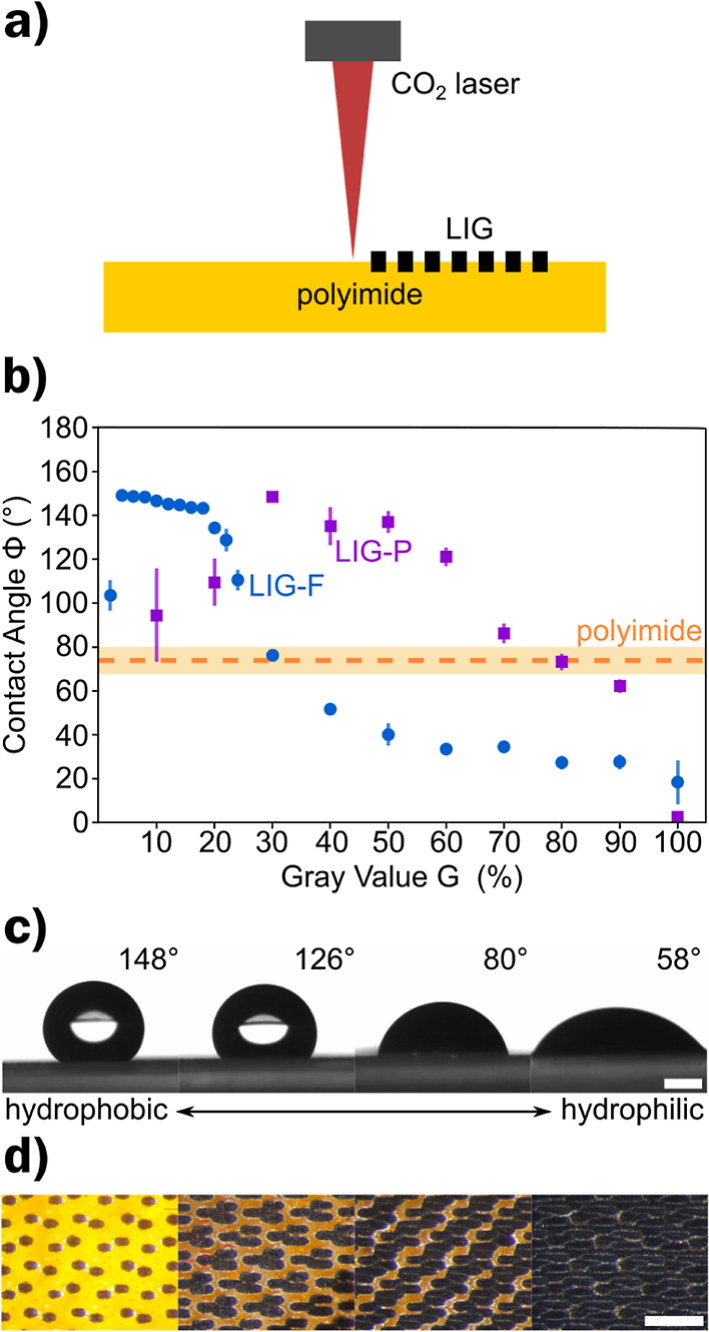
(a) Schematic representation of LIG pattern scribing encoded via the grayscale value *G*. (b) Contact angles of LIG-P and LIG-F scribed at different *G* values and the contact angle of pristine polyimide sheet used as substrate/precursor for LIG patterns. (c) Images of the contact angle measurements (side view) showing the change in wettability for LIG-P *G* at different grayscale values (from left to right: *G* = 30, 50, 70, and 90%), scale bar = 1 mm, and (d) corresponding optical microscope images (top view) of the same LIG-P *G* samples showing the variation in LIG density for different *G* values (scale bar = 500 μm).

The second approach facilitated the change in laser spot density (and therefore laser fluence, i.e., optical energy delivered per surface area unit) determined by the so-called “gray value” of the raster scribing pattern, as depicted in Figure 3a and described in the Materials and Methods. Similar approaches have also been demonstrated by other groups.^27,28,41,49^

One advantage of this approach was that regions with different wettability could be scribed without changing the lasering parameters since the wettability was encoded in the gray value, i.e., the filling density of spots in the pattern. The change of this gray value *G* over the whole possible range (*G* = 0–100%) was investigated to observe the effect on the wettability of both the flat and porous LIG-P and the fibrous LIG-F. Overall, this scribing approach is even faster, cheaper, and more scalable with respect to the purging one.

Figure 3b shows the contact angle Φ of LIG-P and LIG-F scribed with different *G* values and the contact angle of pristine PI with Φ = (74 ± 6)°, which is the substrate over which grayscale patterning of LIG has been performed and therefore represents the *G* = 0% extreme. The PI surface is slightly hydrophilic which is in agreement with the literature.^50,51^ The contact angle of LIG-P increases with increasing *G* values up to a maximum contact angle of Φ = (148 ± 1)° at *G* = 30%. This is slightly lower compared to other publications,^28^ which can be explained by the different laser settings and resulting laser fluence. The increase in contact angle is related to the decrease in wettable PI surface area (yellow areas in Figure S4) and to the increasing density of hydrophobic LIG with the droplet being in a Wenzel state.^34^ At *G* = 30%, the LIG density is high enough to sustain a Cassie–Baxter state with only the LIG being wetted: the droplet is not in contact with the PI surface. With increasing *G* value, the LIG density is increased further (Figure 3d), but the contact angle decreases again until it reaches Φ = 0° at *G* = 100%. The change in contact angle can be probably related to the change in laser fluence, which changes the amount of oxygen present in LIG as described by Nasser et al.^28^ For *G* < 30%, the surface oxygen concentration was low enough to show hydrophobic behavior, while it increased with increasing *G* value and laser fluence. To confirm this change and its origin, XPS spectra were recorded for LIG obtained at different *G* values (Figure S5 and Table S5). The surface oxygen concentration of LIG at *G* = 100% was found to be (5.27 ± 0.05) at %, which agrees with the findings of the LIG-P scribed at ambient conditions from the previous section (Table S2). This is also in agreement with the findings of Nasser et al., where the hydrophilic LIG scribed at high densities showed a surface oxygen concentration of 5.29 at %.^28^ Also, Li et al. reported a similar surface oxygen concentration for LIG scribed in an air-filled chamber.^29^ However, a significantly higher surface oxygen concentration was observed with air assist or in an oxygen-filled chamber.

With decreasing *G*, the surface oxygen concentration of the surface increased to 7 at % for *G* = 70%. These findings are in contrast with the observed wetting behavior. It is possible that the limited spatial resolution of the XPS setup contributed to this discrepancy. While the LIG-P spots were measured, the surrounding PI background was also included in the measurement, which was also observed by Nasser et al.^28^ With decreasing *G* value, the fraction of PI measured increased, and the measured surface oxygen concentration increased too. However, one can expect an interplay of roughness and surface oxygen concentration due to the laser fluence, an effect which is investigated in the next section.

Figure 3c shows the images taken for the contact angle measurements of LIG-P for different *G* values as seen in Figure 3d; one can clearly see the decrease in contact angle with increasing *G* value.

The case of LIG-F is different. LIG-F shows a maximum contact angle of Φ = (149 ± 2)° already at *G* = 4%, with just a little density and coverage of LIG, the wettability is drastically changed with respect to pristine hydrophilic PI (*G* = 0%). This behavior is maintained, with contact angle Φ just slightly decreasing, up to *G* = 20%. At this point, the wetting behavior abruptly changes again at *G* > 30%, LIG-F becomes hydrophilic, and the contact angle drops below the value of PI (Figure 3b).

Like LIG-P, for LIG-F, the hydrophilic state for *G* >20% can be attributed to the (nearly) continuous LIG surface (Figure S6). For *G* < 20%, the same considerations as for LIG-P can be applied for explaining the hydrophobic state.

LIG-F evidenced a distinct metastable Cassie’s–Baxter state; by applying a pressure to the droplet (like dropping it onto the surface from a certain height instead of placing it gently), the wetting was changed into a Wenzel state:^34^ the droplet started wetting the PI surface which resulted in a reduced contact angle (Figure S7).

Overall, distinct and different *G* ranges for hydrophobic and hydrophilic behavior have been evidenced for the cases of LIG-P and LIG-F. Results obtained so far evidenced how, especially when a Cassie–Baxter regime is established, the droplet is in contact only with the terminal, the outermost part of LIG (e.g., the tip of fibers in LIG); so only this part is responsible for the apparent contact angle measured.

Luong et al. showed that hydrophilic LIG can become hydrophobic when transferred to a new substrate.^41^ By turning the LIG upside down, the “deepest” part of the LIG is now on the surface and exposed. It is believed that during laser scribing, the lower, “deep” part of the LIG is protected from atmospheric oxygen by the upper, exposed part of the LIG. This reduced oxygen concentration results in a hydrophobic “deep” LIG. The effect would be similar to the use of local nitrogen purging. Furthermore, the transferred “deep” LIG can be used in combination with the LIG density patterns.^41^ Investigation into the wettability of the deepest part of the LIG were conducted. In order to expose the deepest part of the LIG, the LIG patterns were transferred to an elastomeric matrix by peeling.^37^ The contact angle of the transferred and “leftover” LIG (i.e., the remaining part of the LIG carbon attached to the PI substrate after peeling) was measured. For LIG-P, both the leftover and the transferred LIG-P are superhydrophobic (Φ ∼ 150°) at *G* > 90% (Figure S8). The transferred and leftover LIG-F are superhydrophobic at *G* = 30% [Φ = (155 ± 5)°]; the contact angle slightly decreases at higher *G* values (Figure S9). The transfer exposes a lower part of LIG which has a lower O content, as confirmed by the XPS measurements (Figure S10), which showed an O content of (3.54 ± 0.05) at % for the removed LIG (Table S7), similar to hydrophobic LIG scribed in the local inert atmosphere (Table S2). These results are in agreement with the findings of Luong et al.^41^

### Different Roles of Surface Chemistry and Roughness

**Figure 4:**
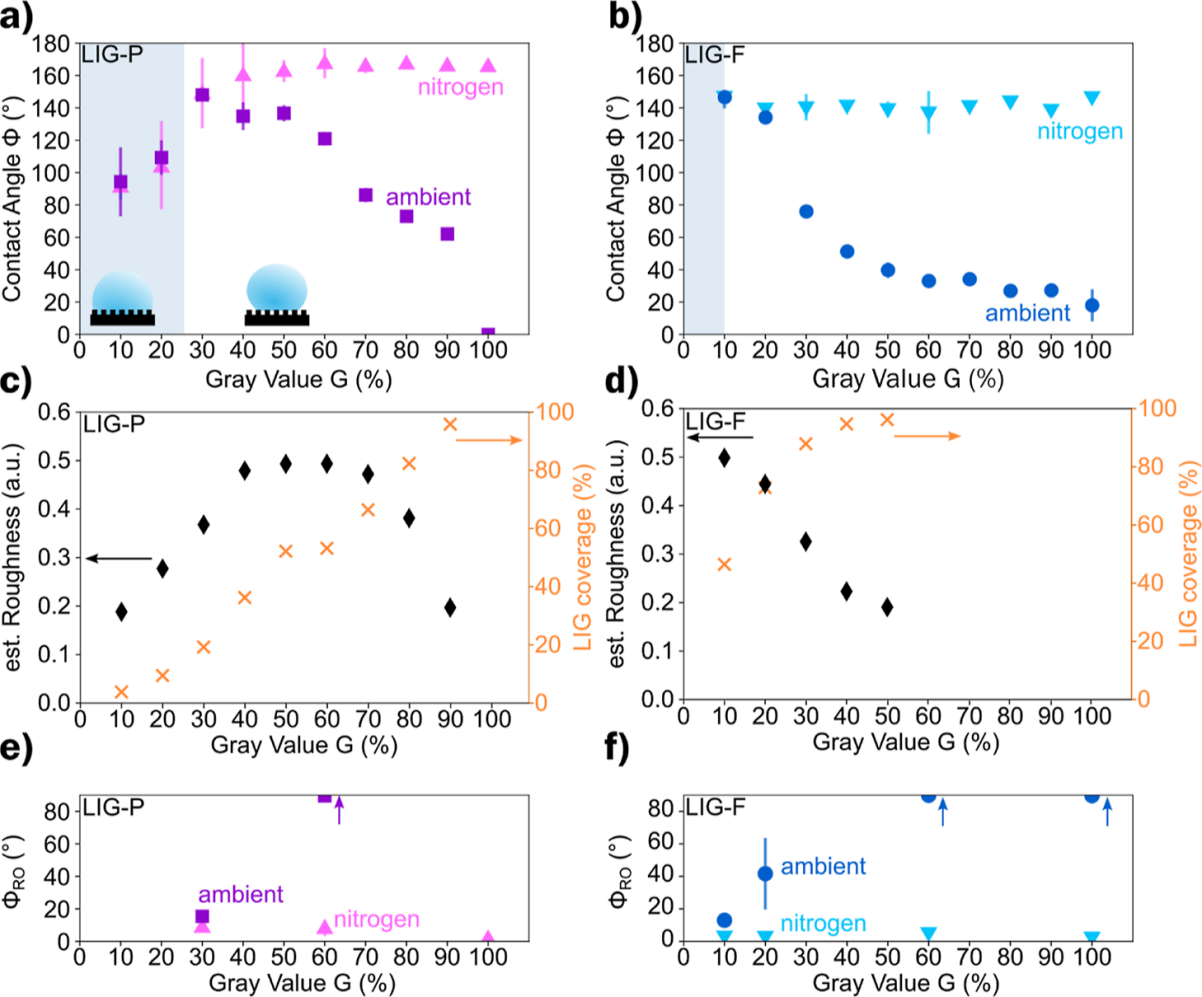
(a,b) Contact angle Φ for LIG-P/LIG-F scribed in ambient and nitrogen environment, shaded area shows Wenzel wetting state. (c,d) Estimated roughness change and LIG coverage of LIG-P/LIG-F at different gray values *G*. (e,f) Roll-off angle Φ_RO_ for LIG-P/LIG-F scribed in ambient and nitrogen environment (values for ambient at *G* = 60% and *G* = 100% are larger than 90° and indicated by the arrow).

To examine the impact of the pattern and roughness on the contact angle Φ, samples of LIG-P and LIG-F with varying gray values (*G* = 10–100%) were scribed with N_2_ purging (*c*_O_2__ = 0% vol) to minimize the presence of surface oxygen groups. The contact angle of ambient and nitrogen-scribed LIG-P samples is shown in Figure 4a. The contact angle at low gray values (*G* = 10–30%) is the same for both types of scribing. This implies that the concentration of surface oxygen is similar in both ambient and nitrogen-scribing conditions. This also confirms that the previous XPS measurements (Table S5) were partially influenced by the LIG density and the extent of exposed PI. For LIG-P with *G* < 30%, a Wenzel state is established, and the apparent contact angle is influenced by the wettability of PI, which was measured to be Φ = (74 ± 6)°. This is highlighted in Figure 4a, where the shaded area represents the Wenzel state. For *G* ≥ 30%, a separation of contact angle values and very different trends are observed for the 2 atm (air, N_2_). The decrease in contact angle of samples scribed in an ambient air atmosphere at increasing *G* indicates that the concentration of surface oxygen increased. Conversely, in the case of nitrogen purging, the contact angle remained at high values (Φ ∼ 150°) and further increased up to *G* = 60%, as shown in Figure 4a. Beyond this point, the LIG-P shows only minor changes in contact angle, implying that the low surface oxygen concentration in the nitrogen-scribed LIG-P was the dominant factor at higher LIG coverage. This increase in contact angle can be attributed to the increased roughness of the LIG surface. A similar phenomenon was observed for LIG-F, as depicted in Figure 4b, despite the fact that the overall trend drastically changed because of the earlier change in roughness and LIG coverage as *G* increased (Figure 4d). This is due to the peculiar long fiber micro-nanostructure of LIG-F.^37,38^ Both air- and nitrogen-scribed LIG-F samples at low *G* exhibit a high contact angle [Φ = (147 ± 2)°] @ *G* = 10%, decreasing to Φ =(134 ± 2)° @ *G* = 20% (Figure 4b) due to the lower roughness (Figure 4d). Differences among the two scribing conditions became apparent at *G* ≥ 20%: the LIG-F coverage is already so high (>80%, Figure 4d) that the roughness plays a negligible role, and the higher surface oxygen concentration is the main contributor to the lower contact angle of ambient scribed LIG-F. Instead, for the nitrogen-scribed LIG-F, the contact angle stayed constant at around Φ ≈ 140° in the range 20% < *G* < 100%.

It can be concluded that the main factor contributing to the hydrophobicity of LIG is indeed its chemical composition (low surface oxygen concentration). Roughness plays only a minor role while coverage is more important. However, not all publications that investigated the wettability of LIG investigated the surface oxygen concentration. However, the data shows that the hydrophobic behavior of LIG with low surface oxygen concentration can be enhanced with patterning, which results in superhydrophobic behavior.^28,42,52,53^

A stricter definition of superhydrophobicity is that the contact angle must exceed 150°, and a small contact angle hysteresis (<10°) and roll-off angle (<5°) must be present.^32^ The measurements of dynamic contact angles and contact angle hysteresis proved to be difficult and did not give reliable results.^54^ Nevertheless, to gain some insight into the dynamic behavior of LIG surfaces, the roll-off angle was measured.

Certain scribing conditions (various *G* values, different atmosphere) per LIG type (LIG-P, Figure 4e and LIG-F, Figure 4f) were chosen to investigate in more detail. For LIG-P and *G* < 30%, the droplet wetted the surface in a Wenzel state which reduced the mobility of the droplet significantly.^34^

Since the focus is on the superhydrophobic behavior, settings of *G* = 30, 60, and 100% were chosen for LIG-P. For LIG-P with a gray value of *G* = 30%, a roll-off angle of Φ_RO_ ≈ 16° was measured when scribed in an ambient atmosphere. In the case of nitrogen-scribed LIG-P with *G* = 30%, a roll-off angle of Φ_RO_ ≈ 10° was measured. The roll-off angle for *G* = 30% for ambient and nitrogen-scribed LIG-P was related to the low surface coverage of LIG. This low coverage favored the Wenzel wetting state and increased the pinning of the droplets. At a higher gray value of *G* = 60%, the ambient scribed LIG-P has a roll-off angle of Φ_RO_ > 90°, while at *G* = 100% no roll-off angle could be measured due to complete wetting. A roll-off angle higher than 90° could not be measured due to the restrictions of the measuring setup. The high roll-off angle could be attributed to the enhanced wetting of the surface and the penetration of the liquid into the porous structure of LIG. This led to a strong anchoring or pinning of the droplets on the surface. For nitrogen-scribed LIG-P, a decrease in roll-off angle to a value of Φ_RO_ = (1.7 ± 0.3)° is observed for *G* = 60 and 100%. This reduction was due to the fact that the droplet was in a Cassie–Baxter state, which hindered it from penetrating the porous structure of the substrate.

LIG-F showed a behavior like LIG-P, an increase in roll-off angle for ambient scribed LIG could be observed for increasing LIG coverage. Values for *G* = 60 and 100% exceeded 90° and could not be measured with the setup. The high roll-off angle could again be attributed to the enhanced wetting of the surface. In the case of nitrogen-scribed LIG-F, a very low roll-off angle of a few degrees with a minimum of Φ_RO_ = (2.4 ± 0.9)° was observed.

This indicates that both LIG-P and LIG-F types showed superhydrophobic behavior (even in the stricter definition with high droplet mobility) when scribed in a nitrogen atmosphere at a high LIG coverage (high gray value *G*). Interestingly, for LIG scribed in an ambient atmosphere at *G* = 30%, the roll-off angle is still relatively high (Φ_RO_ ≈ 16°) compared to the approach by Tittle et al. (Φ_RO_ ≈ 1°).^27^ This indicates that the Cassie–Baxter state is less stable in the case of LIG-P *G* = 30%. This makes sense given that the LIG features are discontinuous and small (resulting in low LIG coverage) compared to Tittle et al. This effect is slightly reduced by the nitrogen purging (Φ_RO_ ≈ 10°) but is still significant.

LIG scribed in an ambient atmosphere showed (super)hydrophilic behavior with either a wicking effect (absorption of the liquid)^55^ or high pinning of the droplets at gray values of *G* > 30%. This effect was also observed by Nasser et al.^28^ The impact of surface roughness only became evident at low LIG coverage (low gray value *G*), as evidenced by a decrease in the contact angle when the droplet is in a Wenzel state.

Unfortunately, all attempts to determine the surface energy of LIG with different techniques have been unsuccessful. To calculate the solid surface energy, the contact angle of different probing liquids is measured and evaluated. Depending on the theory, different liquids with different properties are used.^56−58^ However, the liquids used for different theories (diiodomethane, cyclohexane, chloroform, tetrahydrofuran, glycerol, formamide, and ethylene glycol) were all wetting the surface completely and rendered it impossible to measure a reliable contact angle. This was likely due to the low surface tension and minimal polar component of the liquid–surface interactions. The porous structure of LIG acted like a sponge and fully absorbed the liquid.

### Applications

Both approaches (nitrogen purging and LIG density patterns) can be used in applications that benefit from the self-guiding of liquids (water, biofluids), e.g., “lab-on-chip”.^59^ Since LIG is often used for chemical sensors,^20^ the ability to create patterns with different wettability can be very useful.

**Figure 5:**
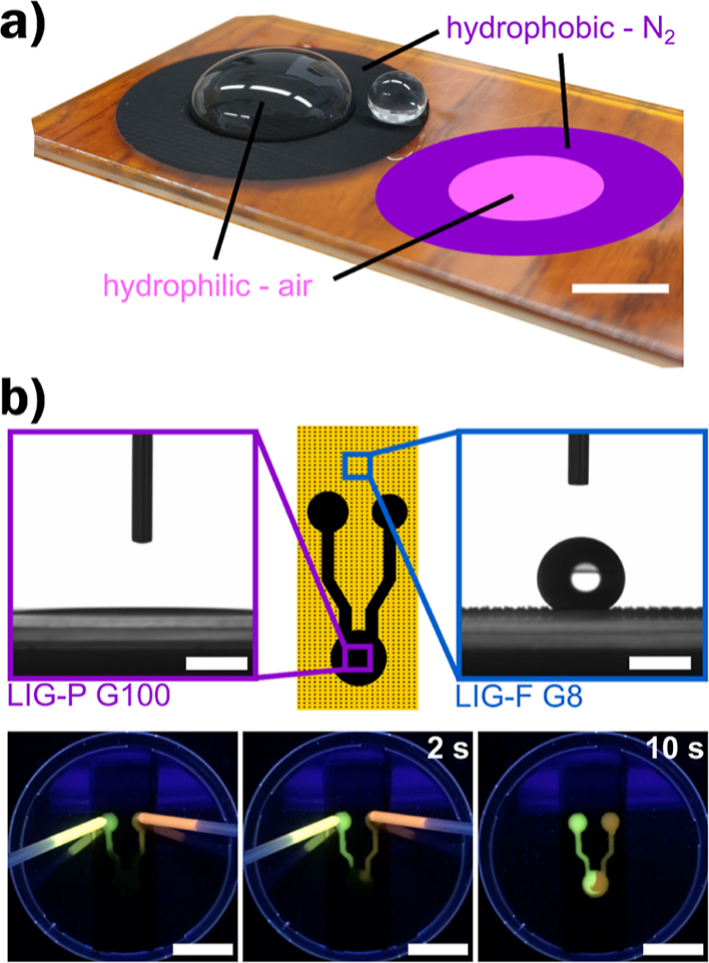
Applications of (a) patterned hydrophobic/hydrophilic LIG surface with a wettability contrast of Φ_in_ – Φ_out_ ≈ 144° confining the water droplet to the inner circle, scale bar = 10 mm. (b) Millifluidics application of high wettability contrast patterning with a grayscale approach; top: design of the demonstrator, featuring superhydrophilic channels and circular chambers (black, LIG-P G100) surrounded by superhydrophobic areas (dotted, LIG-F G8) and corresponding contact angle images; bottom: sequence taken at time *t* = 0, 2, and 10 s showing the self-guiding along channels and mixing of water solutions containing two different fluorescent dyes in patterned LIG channels (a full video is available as Supporting Information).

Thanks to the use of the purging system, a pattern with extreme wettability contrast, as shown in Figure 5a, was produced within a few minutes of laser scribing. The pattern has an outer ring, scribed in a nitrogen atmosphere, with hydrophobic properties, and an inner circle, scribed in air, with hydrophilic properties. Due to the high wettability contrast (Φ_in_ – Φ_out_ ≈ 144°), the droplet placed in the center experiences a distinct confinement, unable to enter the hydrophobic ring region even when the sample is tilted or shaken. A second smaller droplet, placed on the outer hydrophobic region, shows the typical high contact angle shape and could be easily rolled off the surface with only a small tilt or air flow. Such patterns can be used to create a defined volume that can be electrochemically analyzed using the inner circle as an electrode array in LIG-based chemical sensors.

A demonstrator for mixing two liquids in a millifluidic device was created using the LIG density approach (Figure 5b and Video S1). The outer boundary was created by a hydrophobic LIG-F pattern with a low *G* value of *G* = 8%. The inner channels were created by hydrophilic LIG-P patterned with a *G* value of *G* = 100%. The hydrophilic LIG acted as a wick and absorbed the liquid into the mixing chamber. The hydrophobic LIG prevented the liquid from leaving the channels. In this case, water containing two different fluorescent dyes was used to simulate two different liquids and to better demonstrate the phenomenon of mixing under illumination with a UV lamp.

Another application for surfaces with patterned contrast in wettability is fog basking, which is used to capture and collect water droplets from mist, which makes it possible to sustain dry areas suffering from a limited supply of drinking water, with an alternative sustainable water source.^60^ Some fog basking solutions have been inspired by the fascinating example of some Namib Desert beetle species (some species belonging to geni *Stenocara*, *Onymacris*, *Physosterna*).^61,62^ The beetles have patterned elytra with alternating hydrophilic and hydrophobic (waxy) regions. There, the droplets coalesce into larger droplets that are directed through hydrophobic microstructured channels to the beetle’s mouth. The knowledge of patterned wettability via the LIG density patterns was used to investigate the application of LIG/PI surfaces for fog basking.

**Figure 6:**
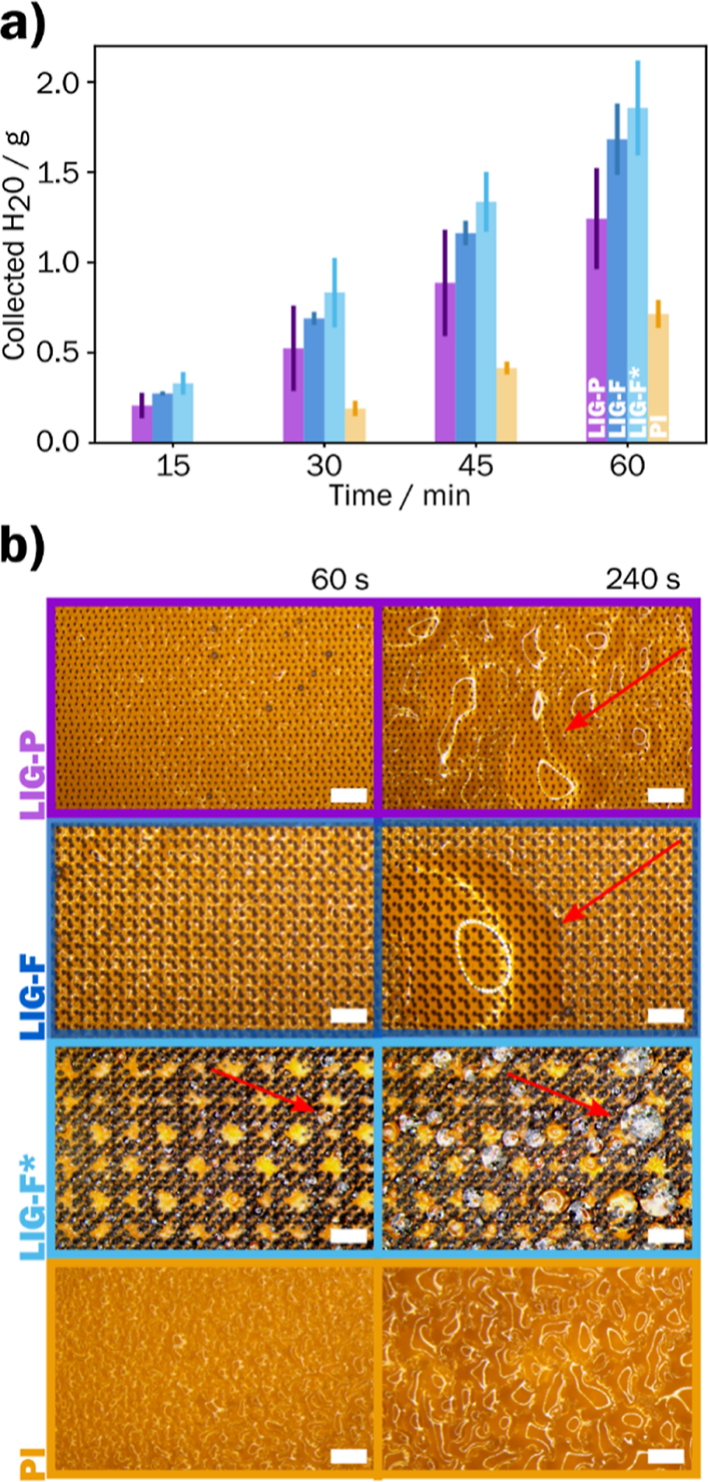
Fog basking results from LIG@PI: (a) Mass of collected water for the different fog collectors in timesteps of 15 min for LIG-P G30, LIG-F G16, LIG-F*, and bare PI; (b) optical microscope images of the fog collectors showing the formation of droplets after 60 and 240 s, red arrows highlighting coagulated water droplets (scale bar = 100 μm).

Various types of hydrophobic LIG on PI with selected settings were created: LIG-P G30 and LIG-F G16 were used due to their near superhydrophobicity. Additionally, a LIG-F* was designed by applying the findings of Garrod et al., where an optimal distance between hydrophilic and hydrophobic centers was found to be 1000 μm.^47^ The CA of LIG-F* was measured to be Φ = (145 ± 4)° (Figure S11). The mass of collected water over time is displayed in Figure 6a for four different fog-collector types: LIG-P, LIG-F, LIG-F*, and bare PI. Over the course of 1 h, LIG-P collected a water mass of *m* = (1.2 ± 0.3) g and LIG-F *m* = (1.7 ± 0.2) g. The special pattern of LIG-F* resulted in an improved collection performance yielding *m* = (1.9 ± 0.3) g over 1 h. Comparing the latter result to the pristine PI, which collected *m* = (0.7 ± 0.1) g of H_2_O, this gives an improved fog collection of 2.6 times.

One reason for the low collection rate of bare PI is the bad performance when it comes to droplet removal from the collection surface. Due to the slightly hydrophilic nature of PI, the droplets need to get much larger before they are affected by gravity and get transported to the bottom where they eventually fall off the collector. In LIG-P and LIG-F, the collected water coalesces into bigger droplets (red arrows) after 240 s, but the surface does not show a distinct hydrophobic behavior because of the meta-stable Cassie’s state, which changes into a Wenzel state (Figure 6b). Since the hydrophobic area (LIG) is smaller than the PI, the droplets are not transitioning into a wetting behavior. LIG-F*, on the other hand, shows a distinct non-wetting behavior from the start and hence, the droplets (red arrow in Figure 6b) are more easily removed from the surface (a recording of this is shown in Video S2).

Although it is very hard to compare the performance of different strategies for fog collection because of different experimental conditions (e.g., wind speed, fog volume, and sample placement),^63,64^ in Table S10, the collection rates taken from recent publications were summarized to provide an idea of their performance. The collection rate of (300 ± 40) mg cm^–2^ h^–1^ achieved by LIG-F* is comparable to other publications listed in Table S10.

## Conclusions

In this study, the wettability of LIG was tuned by different methods, which allowed the creation of patterns with a high wetting contrast. By locally purging nitrogen, it was possible to create LIG samples with tunable wetting behaviors, ranging from hydrophilic to superhydrophobic [Φ = (157 ± 1)°, for LIG-P scribed in nitrogen atmosphere]. The superhydrophobic nature of nitrogen-scribed LIG was maintained for over a year and multiple measurements. Additionally, the combination of scribing LIG density patterns with local nitrogen purging was used to explore the impact of roughness on wettability. The findings indicated that the reduced concentration of surface oxygen was the main factor contributing to the observed wetting behavior. Only when the LIG coverage was low (<30%) did the roughness affect the contact angle as the droplets were in a Wenzel state. The measurement of the roll-off angle showed that nitrogen-scribed LIG at high LIG coverage exhibited superhydrophobic behavior with high droplet mobility. This was demonstrated by the minimum roll-off angle of Φ_RO_ = (1.7 ± 0.3)° recorded for LIG-P. The easy handling of local nitrogen purging and the resulting high wetting contrast can be of interest in micro- and millifluidics and fog basking, as demonstrated. The combination of LIG for self-guidance of liquids, LIG as Joule heaters, and LIG as electrochemical sensors provides a flexible basis for “lab-on-chip” applications based on LIG.^59,65,66^

## Supporting Information Available

Additional experimental schematics, tables of experimental results (Raman, XPS, contact angle), SEM images, and microscope images (PDF)
Millifluidic device for mixing two fluorescent liquids (MP4)
Fog basking device from LIG-F* collecting water (MP4)
