# Supplementary material for: Different Roles of Surface Chemistry and Roughness of Laser-Induced Graphene: Implications for Tunable Wettability
Source: ACS Appl Nano Mater. 2023 Jul 10;6(18):16201–11. doi: 10.1021/acsanm.3c02066 (PMC10526650; doi:10.1021/acsanm.3c02066)
Supplement: Supplementary file 1 — an3c02066_si_001.pdf [file an3c02066_si_001.pdf]

Supporting Information

The Different Roles of Surface Chemistry and Roughness of Laser-Induced Graphene: Implications for Tunable Wettability

Alexander Dallinger[1, +], Felix Steinwender[1, +], Matthias Gritzner[1], Francesco Greco[1, 2, 3, 4, *],

1 Institute of Solid State Physics, NAWI Graz, Graz University of Technology, 8010 Graz, Austria.

2 The Biorobotics Institute, Scuola Superiore Sant’Anna, Viale R. Piaggio 34, 56025 Pontedera, Italy.

3 Department of Excellence in Robotics & AI, Scuola Superiore Sant’Anna, Piazza Martiri della Libertà 33, 56127 Pisa, Italy

4 Interdisciplinary Center on Sustainability and Climate, Scuola Superiore Sant'Anna, Piazza Martiri della Libertà 33, 56127 Pisa, Italy

+ These authors contributed equally to this work.

* Corresponding author: francesco.greco@santannapisa.it

Keywords: laser-induced graphene, tunable wettability, patterning, high contrast, hydrophobic, hydrophilic, superhydrophobicity, millifluidics, fog basking

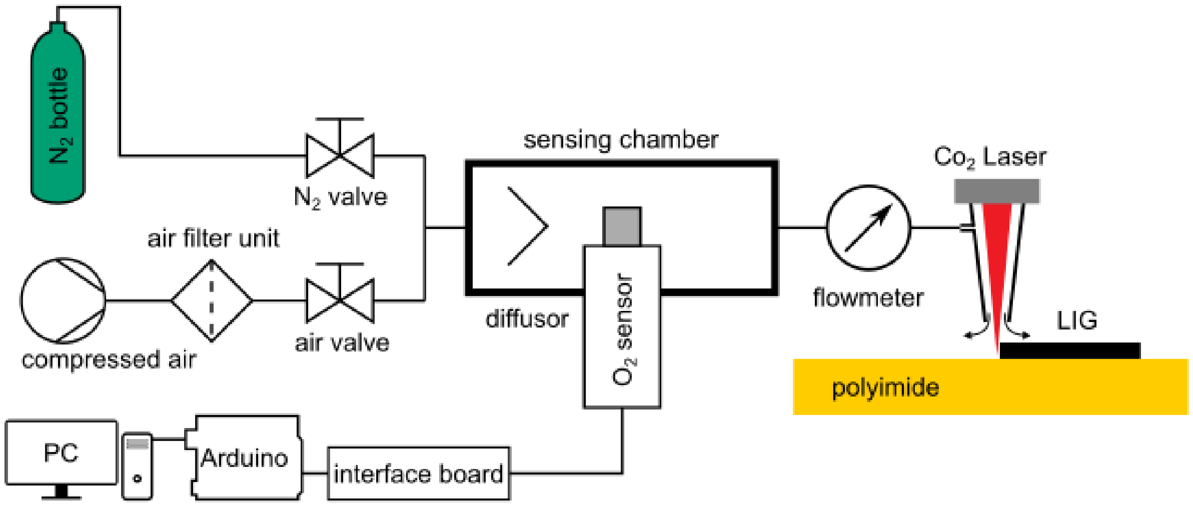

Figure S1: Schematic of nitrogen/air mixture purging and oxygen measurement setup

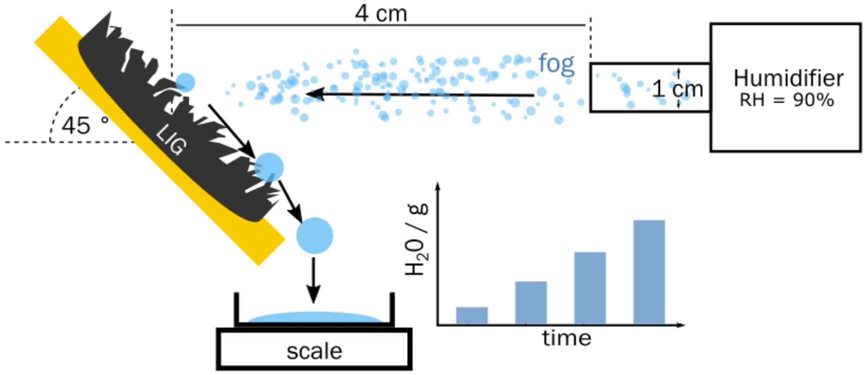

Figure S2: Schematics for the fog basking experiment.

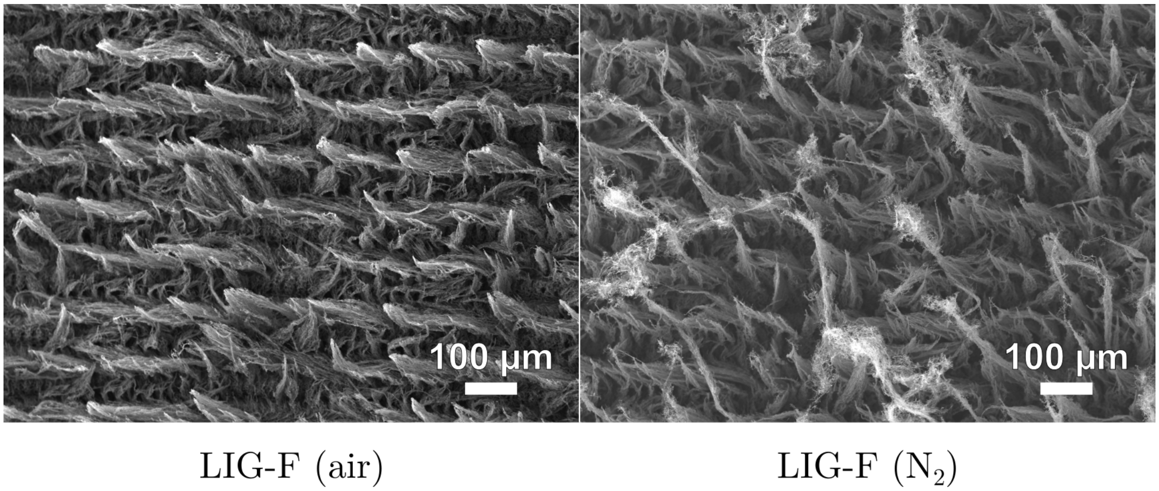

Figure S3: Morphology of both LIG-F variants

Table S1. Values obtained via Raman spectroscopy for LIG-P scribed in different oxygen concentrations of the local atmosphere, I_D/I_G … intensity ratio of D-band to G-band, I_{2D}/I_G … intensity ratio of 2D-band to G-band, v_G … position of G-band, W_D … Full Width Half Maximum of D-band, W_G … Full Width Half Maximum of G-band, W_{2D} … Full Width Half Maximum of 2D-band

**Atmosphere oxygen (%)**	**I_D/I_G**	**I_{2D}/I_G**	**v_G**	**W_D (cm^{-1})**	**W_G (cm^{-1})**	**W_{2D} (cm^{-1})**
20	1.07 ± 0.12	0.28 ± 0.09	1585 ± 5	55 ± 14	51 ± 6	90 ± 30
10	1.05 ± 0.08	0.33 ± 0.09	1587 ± 2	55 ± 13	50 ± 5	81 ± 10
0	1.08 ± 0.11	0.31 ± 0.07	1588 ± 3	60 ± 13	52 ± 6	100 ± 30

Table S2: Atomic weight percent obtained through XPS spectroscopy for LIG-P scribed in different concentrations of atmospheric oxygen

**Atmosphere oxygen (%)**	**C 1s (at%)**	**O 1s (at%)**	**N 1s (at%)**
20	94.14 ± 0.08	5.18 ± 0.05	0.68 ± 0.06
10	95.04 ± 0.09	3.89 ± 0.05	1.08 ± 0.07
0	96.37 ± 0.09	2.65 ± 0.05	0.98 ± 0.08

Table S3: Contact angles of LIG-P scribed with different concentrations of atmospheric oxygen

**Atmosphere oxygen (%)**	**Contact Angle Φ (°)**
0	157 ± 2
5.6	146 ± 8
10.4	128 ± 6
12.2	141 ± 5
14.5	70 ± 10
16.7	56 ± 15
18.2	23 ± 4
20.6	13 ± 7

Greyscale (Dithering) LIG

The following description was taken from the user manual of Universal Laser Systems[1].
"Dithering settings are used when printing grayscale or color bitmapped images such as TIF, JPG and BMP formatted images. Since the laser system is essentially a black and white printer (black turns the laser ON and white turns the laser OFF), and if you choose the correct settings, the driver will automatically convert the grayscale or color bitmap into a 1-bit "halftoned" black and white image. This process is very similar to how newspaper photographs, as well as laser printer photographs, are printed. For a more detailed explanation of the terms "grayscale," "bitmap," "halftone" or "dither," please refer to the "Graphic Software Setup" section in this manual."

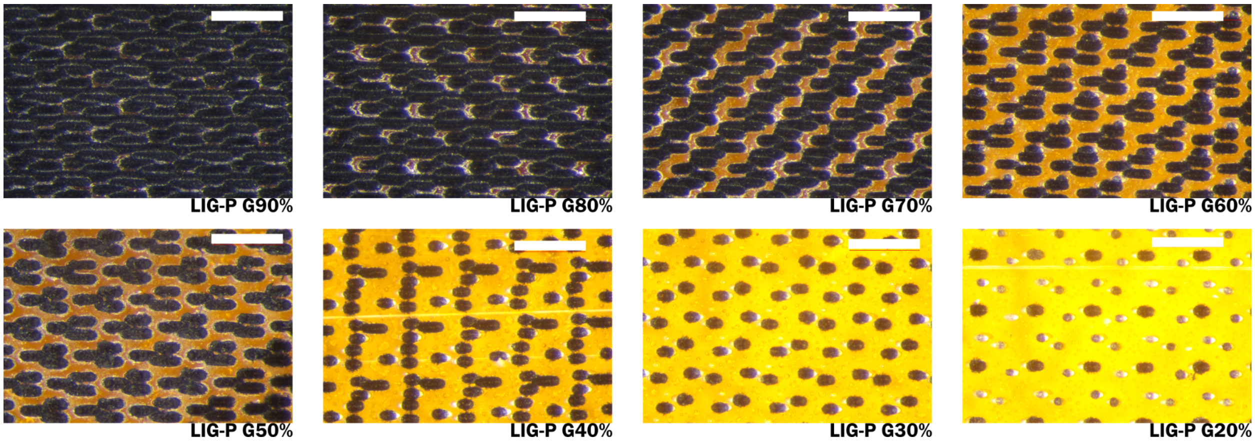

Figure S4: Surface of LIG-P/PI at different gray scale values from G = 90% to 20%, scale bar = 500 µm

[1] VLS 2.30 - ULS User Guide, © 2008 Universal Systems, Inc.

Table S4: LIG-P CA corresponding to Figure 3b)

Gray Value G (%)	Contact Angle Φ (°)
100	0 ± 0
90	62 ± 3
80	73 ± 4
70	86 ± 4
60	121 ± 4
50	137 ± 5
40	135 ± 9
30	148 ± 1
20	110 ± 10
10	95 ± 21

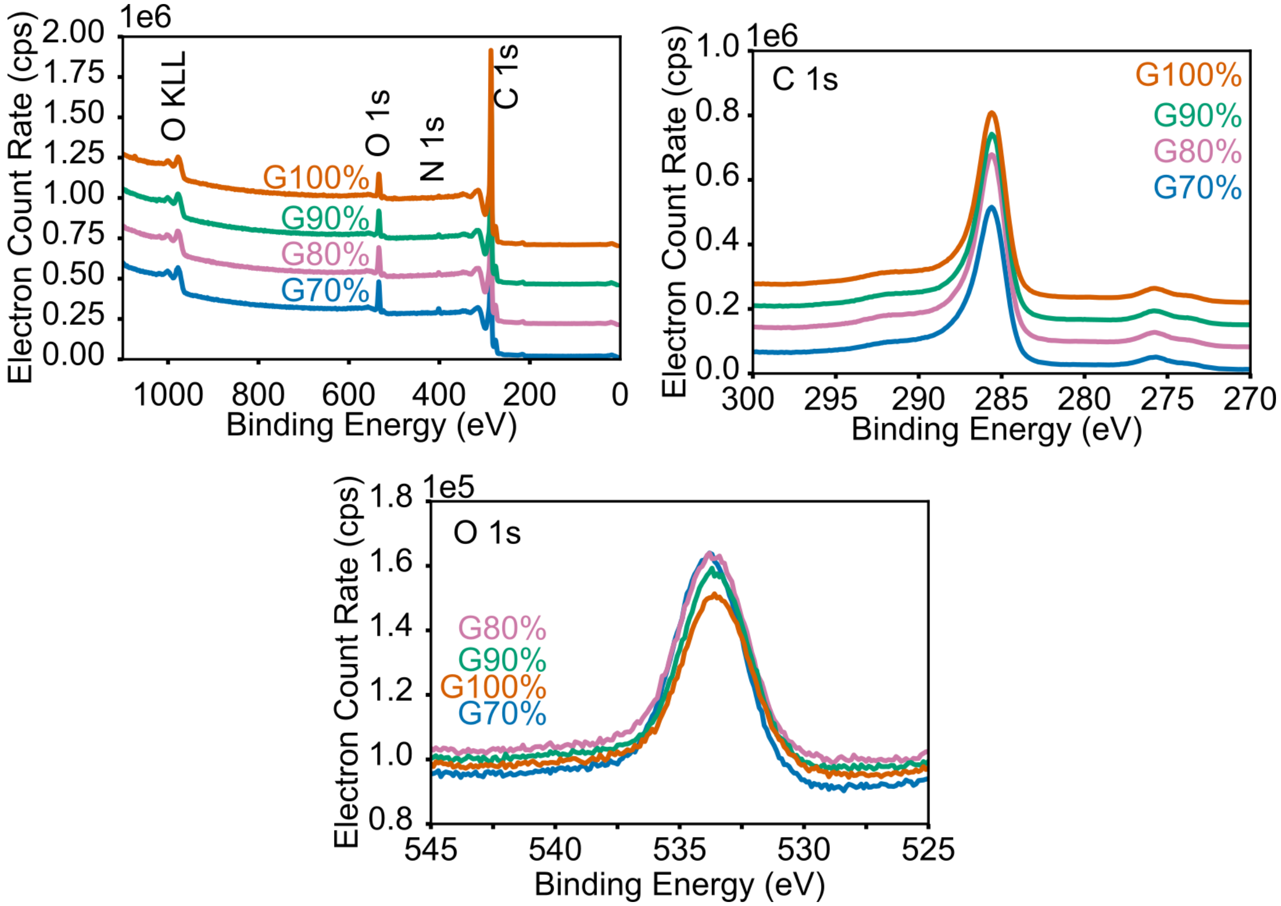

Figure S5: XPS Spectra of LIG-P with different gray values from 70% - 100%

Table S5: Quantification of XPS measurement results for LIG-P G100% to G70%

	**LIG-P G100%**	**LIG-P G90%**	**LIG-P G80%**	**LIG-P G70%**
C 1s (at%)	94.35 ± 0.07	93.65 ± 0.09	93.03 ± 0.09	91.23 ± 0.09
O 1s (at%)	5.27 ± 0.05	5.46 ± 0.06	6.1 ± 0.06	7.09 ± 0.06
N 1s (at%)	0.38 ± 0.05	0.88 ± 0.07	0.87 ± 0.08	1.68 ± 0.08

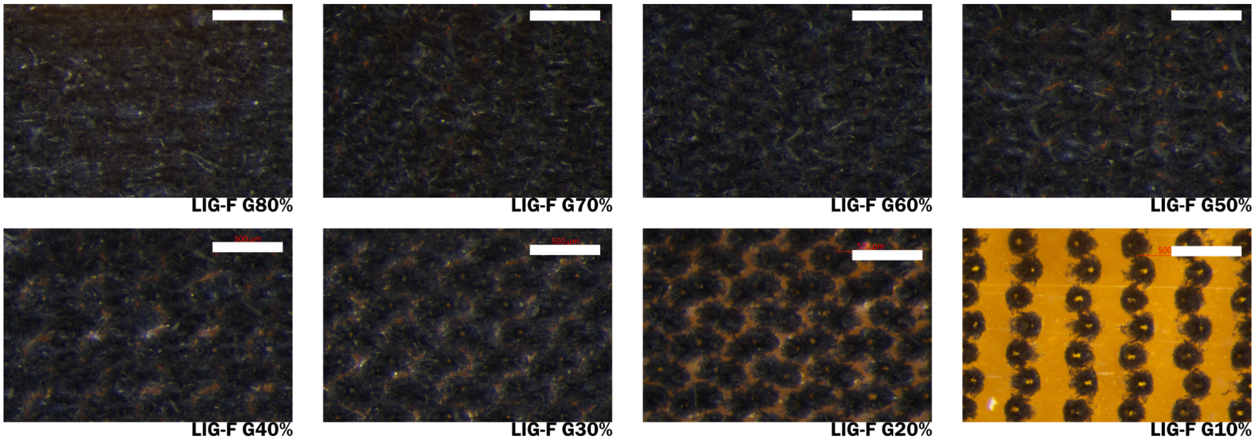

Figure S6: Surface of LIG-F/PI at different gray scale values from G = 80% to 10%, scale bar = 500 µm

Table S6: LIG-F CA corresponding to Figure 3b)

**Gray Value G (%)**	**Contact Angle Φ (°)**
100	20 ± 10
90	28 ± 3
80	27 ± 3
70	34 ± 2
60	33 ± 2
50	40 ± 5
40	52 ± 2
30	76 ± 3
20	134 ± 2
10	147 ± 2

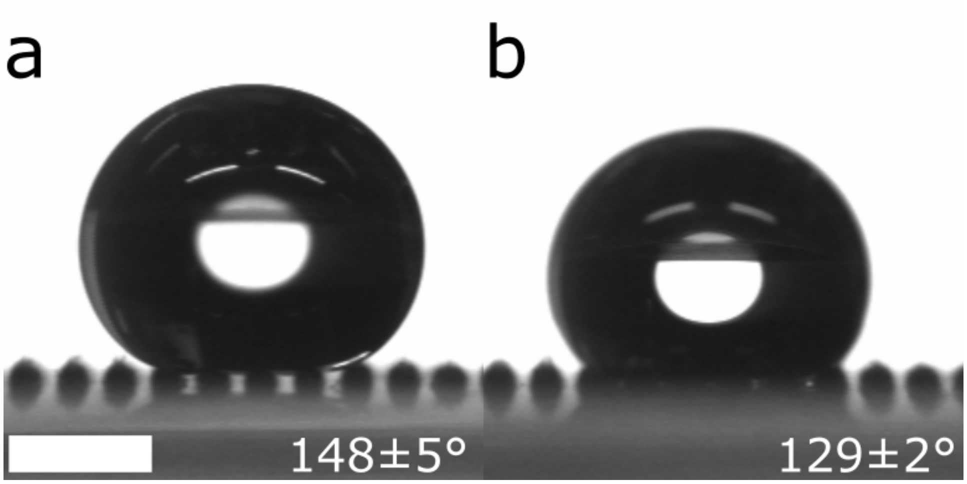

Figure S7: Change of a metastable Cassie-Baxter state into a Wenzel state with reduced contact angle, scale bar = 1mm.

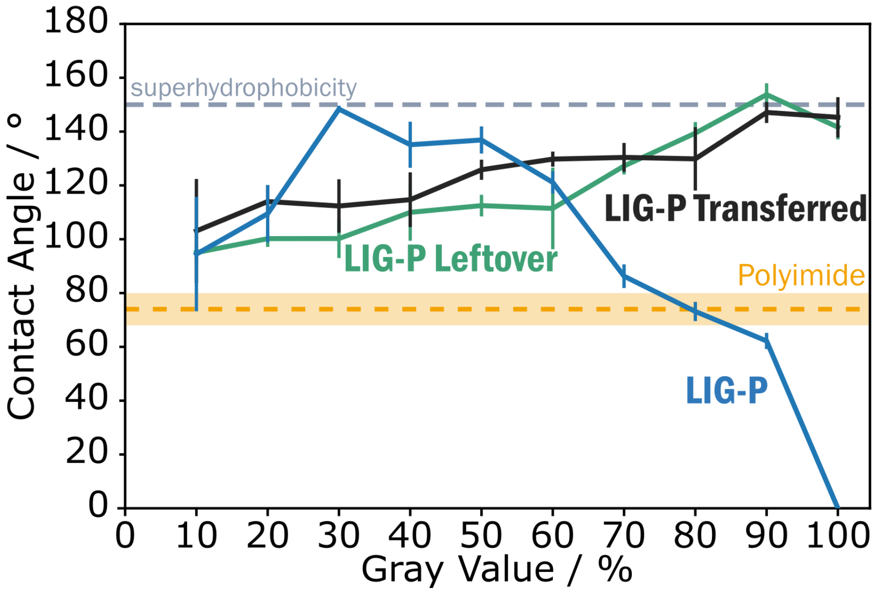

Figure S8: Wettability of LIG-P, LIG-P transferred and LIG-P leftover.

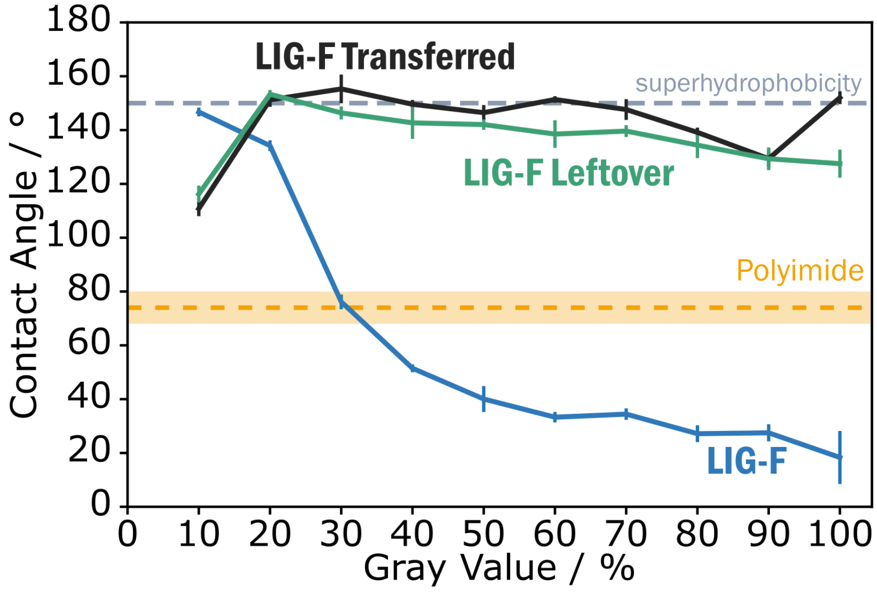

Figure S9: Wettability of LIG-F, LIG-F transferred and LIG-F leftover.

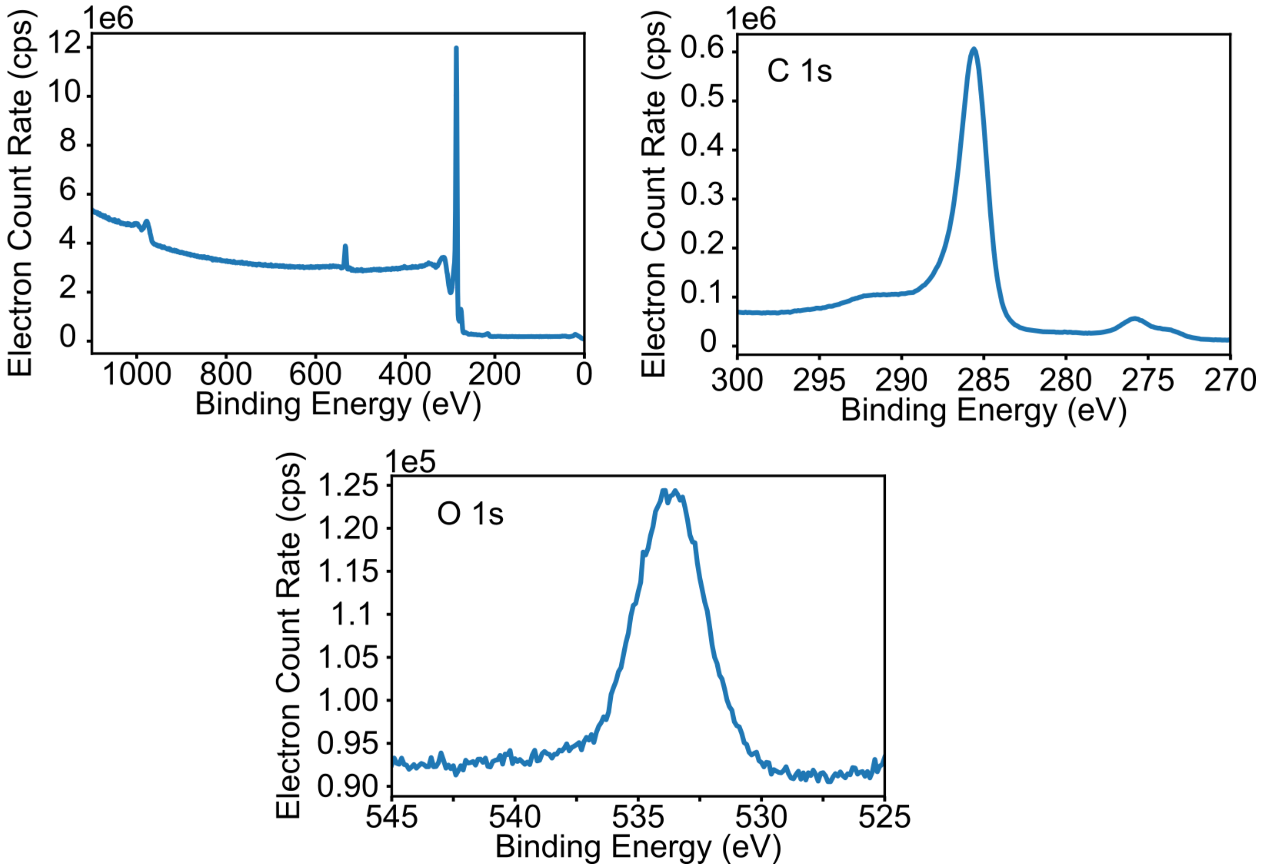

Figure S10: XPS Spectrum of removed LIG-P showing the chemical composition of hydrophobic LIG. The leftover LIG was measured and assumed to be the same as the transferred and exposed one.

Table S7: Quantification of XPS measurement result for removed LIG-P

	**Removed LIG-P**
C 1s (at%)	96.17 ± 0.08
O 1s (at%)	3.54 ± 0.05
N 1s (at%)	0.29 ± 0.06

Table S8: LIG-P values from Figure 3. CA … contact angle, Φ_{RO} … roll-off angle

**Greyvalue (%)**	**CA Φ(°)**	**CA Φ_{N2} (°)**	**Roughness (a.u.)**	**Coverage (%)**	**Φ_{RO} (°)**	**Φ_{RO_N2}(°)**
100	0 ± 0	166 ± 5	—	—	—	1.7 ± 0.3
90	62 ± 3	166 ± 5	0.20	95.9	—	—
80	73 ± 4	167 ± 2	0.38	82.4	—	—
70	86 ± 4	166 ± 3	0.47	66.4	—	—
60	121± 4	168 ± 6	0.49	53.2	>90°	8.1 ± 4.9
50	137 ± 5	163 ± 3	0.49	52.2	—	—
40	135 ± 9	160 ± 4	0.48	36.3	—	—
30	148 ± 1	150 ± 20	0.37	19.3	16 ± 3	10.1 ± 1.3
20	110 ± 10	105 ± 7	0.28	9.5	—	—
10	95 ± 20	91 ± 5	0.19	3.8	—	—

Table S9: LIG-F values from Figure 3. CA … contact angle, Φ_{RO} … roll-off angle

**Greyvalue (%)**	**CA Φ(°)**	**CA Φ_{N2} (°)**	**Roughness (a.u.)**	**Coverage (%)**	**Φ_{RO} (°)**	**Φ_{RO_N2}(°)**
100	20 ± 10	147 ± 4	—	—	>90°	2.4 ± 0.8
90	28 ± 3	139 ± 1	—	—	—	—
80	27 ± 3	144 ± 3	—	—	—	—
70	34 ± 2	141 ± 3	—	—	—	—
60	33 ± 2	137 ± 2	—	—	>90°	5.1 ± 1.2
50	40 ± 5	139 ± 1	0.19	96.2	—	—
40	52 ± 2	141 ± 4	0.22	94.8	—	—
30	76 ± 3	140 ± 3	0.33	87.9	—	—
20	134 ± 2	140 ± 1	0.44	72.9	42 ± 22	2.8 ± 0.5
10	146 ± 2	147 ± 1	0.50	46.5	13 ± 2	3.0 ± 0.8

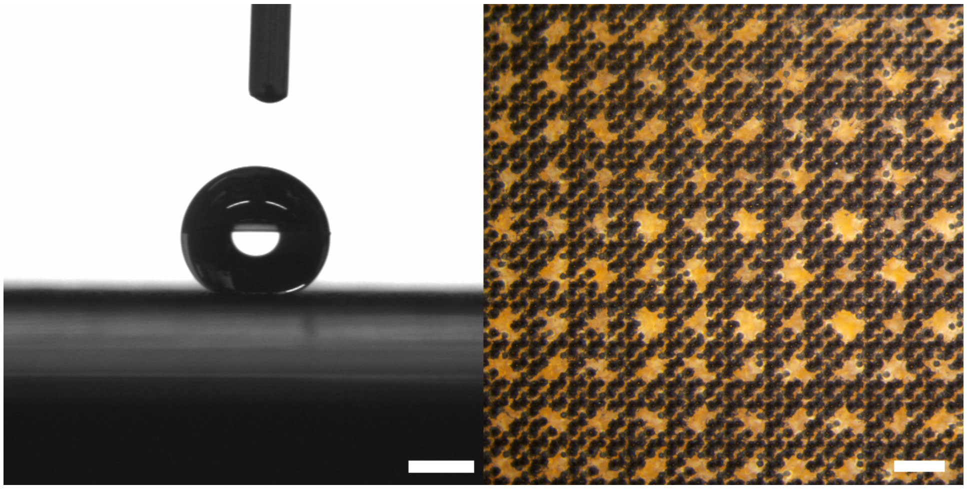

Figure S11: (left) Contact angle image of LIG-F* with Φ = (144.6 ± 3.9)°; (right) microscope images of the LIG-F* sample, scale bar = 1 mm

Table S10: Comparison of different fog basking publications and their fog basking collection rate.

Type	Collection Rate [mg cm^{-2} h^{-1}]	Reference
LIG-P on PI	200 ± 50	this work
LIG-F on PI	270 ± 30	this work
LIG-F* on PI	300 ± 40	this work
LIG-VEC on PI	310 ± 30	this work
Patterned LIG on PI-Paper	3280	1
Janus fiber (PS & CA)	71	2
Raschel mesh (PA6)	64	3
Micro-patterned copper oxide nanoneedle	66 ± 4	4
Hierarchically-textured surface (PP)	1029 ± 108	5
Cicada wing surface	39.6	6
Nanoscale PDA coated SU-8 bumps	97	7
Raschel meshes (hydrophobic coating)	3490	8
SLA 3D printed cones	2680	9
Janus Fabric with Asymmetric Wettability	224.7	10

References

(1) Wang, Y.; Wang, G.; He, M.; Liu, F.; Han, M.; Tang, T.; Luo, S. Multifunctional Laser-Induced Graphene Papers with Combined Defocusing and Grafting Processes for Patternable and Continuously Tunable Wettability from Superlyophilicity to Superlyophobicity. *Small* **2021**, *17* (42), 2103322. https://doi.org/10.1002/smll.202103322.

(2) Knapczyk-Korczak, J.; Zhu, J.; Ura, D. P.; Szewczyk, P. K.; Gruszczyński, A.; Benker, L.; Agarwal, S.; Stachewicz, U. Enhanced Water Harvesting System and Mechanical Performance from Janus Fibers with Polystyrene and Cellulose Acetate. *ACS Sustainable Chem. Eng.* **2021**, *9* (1), 180–188. https://doi.org/10.1021/acssuschemeng.0c06480.

(3) Knapczyk-Korczak, J.; K. Szewczyk, P.; P. Ura, D.; Berent, K.; Stachewicz, U. Hydrophilic Nanofibers in Fog Collectors for Increased Water Harvesting Efficiency. *RSC Advances* **2020**, *10* (38), 22335–22342. https://doi.org/10.1039/D0RA03939J.

(4) Sharma, V.; Yiannacou, K.; Karjalainen, M.; Lahtonen, K.; Valden, M.; Sariola, V. Large-Scale Efficient Water Harvesting Using Bioinspired Micro-Patterned Copper Oxide Nanoneedle Surfaces and Guided Droplet Transport. *Nanoscale Adv.* **2019**, *1* (10),

4025–4040. https://doi.org/10.1039/C9NA00405J.
(5) Raut, H. K.; Ranganath, A. S.; Baji, A.; Wood, K. L. Bio-Inspired Hierarchical Topography for Texture Driven Fog Harvesting. *Applied Surface Science* **2019**, *465*, 362–368. https://doi.org/10.1016/j.apsusc.2018.09.134.
(6) Xie, H.; Huang, H.-X.; Mi, H.-Y. Gradient Wetting State for Droplet Transportation and Efficient Fog Harvest on Nanopillared Cicada Wing Surface. *Materials Letters* **2018**, *221*, 123–127. https://doi.org/10.1016/j.matlet.2018.03.066.
(7) Moazzam, P.; Tavassoli, H.; Razmjou, A.; Warkiani, M. E.; Asadnia, M. Mist Harvesting Using Bioinspired Polydopamine Coating and Microfabrication Technology. *Desalination* **2018**, *429*, 111–118. https://doi.org/10.1016/j.desal.2017.12.023.
(8) Rajaram, M.; Heng, X.; Oza, M.; Luo, C. Enhancement of Fog-Collection Efficiency of a Raschel Mesh Using Surface Coatings and Local Geometric Changes. *Colloids and Surfaces A: Physicochemical and Engineering Aspects* **2016**, *508*, 218–229. https://doi.org/10.1016/j.colsurfa.2016.08.034.
(9) Peng, L.; Chen, K.; Chen, D.; Chen, J.; Tang, J.; Xiang, S.; Chen, W.; Liu, P.; Zheng, F.; Shi, J. Study on the Enhancing Water Collection Efficiency of Cactus- and Beetle-like Biomimetic Structure Using UV-Induced Controllable Diffusion Method and 3D Printing Technology. *RSC Advances* **2021**, *11* (24), 14769–14776. https://doi.org/10.1039/D1RA00652E.
(10) Zhu, R.; Liu, M.; Hou, Y.; Zhang, L.; Li, M.; Wang, D.; Wang, D.; Fu, S. Biomimetic Fabrication of Janus Fabric with Asymmetric Wettability for Water Purification and Hydrophobic/Hydrophilic Patterned Surfaces for Fog Harvesting. *ACS Appl. Mater. Interfaces* **2020**, *12* (44), 50113–50125. https://doi.org/10.1021/acsami.0c12646.
